# *Drosophila* attraction, colonization, contagion, and mortality by *Pseudomonas* spp. and toxicity of their biosurfactants

**DOI:** 10.1007/s00253-025-13518-x

**Published:** 2025-06-04

**Authors:** Argyro Tsipa, Maria Pettemereidi, Constantina K. Varnava, Izel Ungor, Eftychia Fragkou, Yiorgos Apidianakis

**Affiliations:** 1 Department of Civil and Environmental Engineering, University of Cyprus, 75 Kallipoleos, 1678 Nicosia, Cyprus; 2 Nireas International Water Research Centre, University of Cyprus, 1678 Nicosia, Cyprus; 3 Department of Biological Sciences, University of Cyprus, 1678 Nicosia, Cyprus

**Keywords:** Oily wastewater bioremediation, Biocontrol agents, Circular bioeconomy, Environmental resilience

## Abstract

**Abstract:**

Oil bioremediation may be achievable via *Pseudomonas* spp. leading to low-cost biosurfactant (BSF) production, but the environmental impact is unclear. Here, we studied *P. aeruginosa* PA14 and PAO1; *P. putida* mt-2 and F1; and *P. citronellolis* 620C, P3B5, and SJTE-3, for their ability to degrade oily wastewater (OW), produce BSFs, and impact the model insect, *Drosophila melanogaster*. Biodegradation was > 86% by day 1 and > 93% by day 7, while BSF production was > 200 mg/L by day 1 and > 400 mg/L by day 7 for all strains. *P. aeruginosa* PAO1 and PA14 produce rhamnolipids and glycolipopeptides, respectively. *P. putida* mt-2 and F1 formed glycolipopeptides and glycolipids, respectively*. P. citronellolis* P3B5 and SJTE-3 yielded glycolipids, whereas 620C produced lipopeptides. Strikingly, *Drosophila* was mostly attracted to food contaminated with any of the *P. aeruginosa* strains or *P. putida* mt-2, which were the most virulent. To the contrary, *Drosophila* was repelled from food containing the low in virulence *P. putida* F1 or any of the *P. citronellolis* strains. All strains exhibited high ability to colonize *Drosophila* and disperse from fly to fly, but the colonization and contagion extend by *P. aeruginosa* strains were slightly higher. Moreover, the virulence of *Pseudomonas* spp. aligned with the toxicity of their BSFs. BSFs produced by *P. aeruginosa* were the most toxic, followed by *P. putida* and *P. citronellolis*, indicating a correlation between BSF toxicity and microbial origin. We concluded that *P. citronellolis* strains and their BSFs are relatively innocuous to the fly populations, yet highly potent in biodegrading OW.

**Key points:**

• *>93% biodegradation of oily wastewater by all Pseudomonas spp. strains by day 7*

• *The virulence of Pseudomonas spp. aligns with the toxicity of their BSFs*

• *P. citronellolis strains and their BSFs are more innocuous to Drosophila than those of P. putida and P. aeruginosa*

**Supplementary Information:**

The online version contains supplementary material available at 10.1007/s00253-025-13518-x.

## Introduction

Synthetic surfactants are commonly used for the enhancement of biological performance, maintenance of physical stability, and preparation of agrochemical formulations, but they are toxic to ecosystems. Towards green agrichemicals, these surfactants could be replaced by their bio-based counterparts (Silva et al. 2024). Similar to synthetic surfactants, bio-based surfactants are amphiphilic compounds that reduce the surface/interface tension between liquid and solid compounds (Das and Mukherjee 2007). Bio-based surfactants can be microbial or plant-derived or synthesized using natural extracts. These surfactants are eco-friendly and biodegradable compared to synthetic surfactants. Among them, microbial surfactants, the so-called biosurfactants (BSFs), could be used as biocontrol agents in agriculture (Sachdev and Cameotra 2013; Eras-Muñoz et al. 2022; Gayathiri et al. 2022), among several other applications in life sciences (e.g., antimicrobial and antibiofilm agents), industry (e.g., in petroleum, food, and textile manufacturing), and environment (e.g., wastewater treatment, bioremediation, oil spill decontamination, and recovery) (Mulligan 2005; Markande et al. 2021; Sarubbo et al. 2022). BSFs are barely toxic to vertebrates and can be easily produced (Parthipan et al. 2018). In agriculture, BSFs can improve soil quality, promote plant growth, and stimulate the plant immune system and defense genes leading to their protection against hostile activities, thus, acting as biocontrol agents due to their antimicrobial, antifungal, antinsecticidal, antilarvicidal, antimosquitocidal, emulsification, and antibiofilm formation properties (Silva et al. 2024). There are several studies about BSFs, mainly glycolipids and lipopeptides, primarily coming from *Bacillus* and *Pseudomonas* spp. acting as biocontrol agents against agricultural pests, as well as in insects that transmit diseases to humans (Edosa et al. 2018; Fernandes et al. 2024).

One of the main limitations of scalability and broad availability of BSFs is the high cost of substrates which accounts for up to 50% of the final bioproduction costs (Varnava et al. 2024a). A strategy to overcome this restriction is the use of low (even no)-cost alternative substrates. Such substrate could be oily wastewater (OW), including vegetable, petroleum, and non-petroleum OW streams. In the oil and gas (O & G) industry, drilling waste and wastewater (DWW) is the second largest in volume generated waste stream, posing notable waste management issues (Onwukwe and Nwakaudu 2013). Drilling wastewater (DW) contains many pollutants such as hydrocarbons, which, unless properly managed, result in contamination of the natural ecosystems (Tsipa et al. 2021a). Advanced physicochemical and thermal processes are often used for OW remediation. However, they are cost and energy-intensive, producing secondary toxic products (Miklos et al. 2018). On the other hand, the use of microorganisms in remediation, which is called bioremediation, is an eco-friendly, nature-based alternative considered a basic strategy for managing oil-polluted areas and OW (Putatunda et al. 2019; Adetunji and Olaniran 2021; Kumar et al. 2022). This approach has, also, been used for DW biodegradation resulting in BSF production using *Pseudomonas citronellolis* strains (Koutinas et al. 2021; Tsipa et al. 2021a, b; Varnava et al. 2024a, b). However, there is an open scientific question on whether these DW-produced BSFs could be used as biocontrol agents in agriculture among other applications.

Microbes could also be used as potent biocontrol agents in agriculture. Plant diseases caused by insects and pests significantly reduce seed quality and germination, thus restricting the potential plant yield and development. Microorganisms can act through mechanisms of parasitism, antibiosis, volatile toxic compounds and mycolytic enzymes’ secretion, and competition for nutrients and space against hostile parties. Among those beneficial microorganisms, *Trichoderma* fungi and *Pseudomonas* and *Bacillus* bacteria are the most promising biocontrol agents acting against a wide range of plant pathogens (Ritika and Utpal 2014; Mnif and Ghribi 2015).

Despite the numerous studies performed on the effect of specific microbes and BSFs against insects and pests, the antinsecticidal effect of bacteria of the same genus and their derived BSFs may vary significantly. And, despite the lower production costs of the use of OW, such as DW, to produce BSFs, the effect of those extracted BSFs on insects and pests remains unknown. Furthermore, studies using microbes and BSFs against flies as representative vectors remain scarce (Assie et al. 2002; Mohammed Mostakim 2012; Mnif and Ghribi 2015; Ramesar and Hunter 2023), and very little has been done to shed light on their potential on fly attraction, colonization, and longevity (Kapsetaki et al. 2014).

In this study, the biodegradation potential of *Pseudomonas* spp. and their ability to form BSFs using DW is assessed as an alternative production way to reduce the operational cost. We chose *P. aeruginosa* and *P. putida* strains because the corresponding species can be found in the soil and may degrade several pollutants (Schroth et al. 2018; Vodovar et al. 2005; Kivisaar 2020; Hu et al. 2023). *P. aeruginosa* is known to produce rhamnolipids (Maier and Soberón-Chávez 2000), while *P. putida* is a species which can produce lipopeptides, glycolipids, and phospholipids (Bernat et al. 2019; Cesa-Luna et al. 2023; Janek et al. 2013; Kuiper et al. 2004; Nanganuru and Korrapati 2012; Petrikov et al. 2013; Raaijmakers et al. 2010; Rokni-Zadeh et al. 2012, 2013; Tuleva et al. 2002; Zhou et al. 2024). *P. citronellolis* is the third chosen species of the current study which is also found in soil (Bhattacharya et al. 2003). *P. citronellolis* 620C can degrade DW producing a lipopeptide BSF (Varnava et al. 2024a).

Insights on fly attraction, colonization, and longevity due to exposure to *Pseudomonas* spp. and their BSFs are determined. These insights indicate the effect of those microorganisms and their relevant bio-products on natural ecosystems in which flies live and spread. The effect of microorganisms on flies implies to which extent these microorganisms may be used in biotechnological applications and as biocontrol agents. In addition, the BSFs used come from the OW bioremediation by *Pseudomonas* spp. Therefore, the effect of those BSFs on flies indicates the broadness of their applicability since their toxicity will be determined.

## Materials and methods

### Bacterial strains and culture conditions

In this study, seven bacterial strains were used: *P. aeruginosa* PA14 (from the University of California Berkeley Plant Pathology laboratory under no. PA14), PAO1 (available from the American Type Culture Collection-ATCC under no. 15692); *P. putida* mt-2 (ATCC 33015), F1 (ATCC 700007) (Panayidou et al. 2020); *P. citronellolis* P3B5, SJTE-3, 620C. *P. citronellolis* 620C strain was isolated from an OW stream, drill cutting fluid, and activated sludge (Tsipa et al. 2021a). The strain P3B5 was isolated from the phyllosphere of basil plants (Remus-Emsermann et al. 2016) and obtained by the School of Biological Sciences of the University of Canterbury Warehouse, New Zealand. The strain SJTE-3 (available from the China General Microbiological Culture Collection Center under no. 12720) was isolated from activated sludge of a wastewater treatment plant in China (Zheng et al. 2016) and obtained by the Laboratory of Microbial Metabolism and School of Life Sciences & Biotechnology, Shanghai Jiaotong University, China.

For fly experiments, bacterial strains, stored as 25% glycerol stocks at − 80 °C, were streaked on agar plates (32 g of LB agar in 1 L of ddH_2_O) and incubated at 37 °C for 16 h. Single colonies were placed in 3 mL of LB broth medium (20 g LB broth per 1 L of ddH_2_O) and shaken overnight for 16 h at 200 rpm and 37 °C. Thirty microliters of the overnight culture was added in 3 mL of fresh LB medium (OD_600nm_ ~ 0.05) and incubated at 200 rpm and 37 °C for 4–5 h to OD_600nm_ = 3 (for fly survival to infection by feeding) or 7–8 h to OD_600nm_ = 5 (for fly attraction assessment).

For the OW biodegradation potential of the bacterial strains, pre-cultures and sub-cultures were prepared as described by Varnava et al. (2024a). Sterilized slightly modified M9 minimal medium (67.82 g/L Na_2_HPO_4_, 30 g/L KH_2_PO_4_, 30 g/L NaCl, 10 g/L NH_4_Cl, and 0.02% MgSO_4_ in ddH_2_O water) supplemented with 1% v/v OW, provided by Innovating Environmental Solutions Center (IESC Ltd., Limassol, Cyprus), was used. The pre-culture and sub-culture flasks were filled with medium up to one-fifth of their volume to ensure sufficient oxygen availability to the microorganism. All biodegradation experiments were performed in triplicates.

### Phylogenetic tree construction

Molecular Evolutionary Genetics Analysis software 11 (MEGA11) was used for phylogenetic tree construction. Firstly, 16S ribosomal RNA (rRNA) sequences of the microorganisms used in this study were obtained in FASTA format from the National Center for Biotechnology Information (NCBI) GenBank database using their accession numbers. The 16S rRNA gene of the *Escherichia coli* K12 strain (with accession number U00096.3) was used as reference. Then, multiple sequence alignment (MSA) was performed using the obtained DNA FASTA sequences. Then, the FASTA file containing aligned sequences was converted to a MEGA format file. MEGA 11 software tool was used to construct the neighbor-joining tree from the MEGA format file. Bootstrap analysis with 1000 replicates was performed to assess the reliability of the branches in the phylogenetic tree. Also, the p-distance model was selected for calculating genetic distances between sequences.

### Biosurfactant extraction and partial purification

The BSFs were extracted and partially purified from the bacterial cultures according to the protocol described by Varnava et al. (2024a). Briefly, the bacterial cultures were subjected to centrifugation at 4 °C for 30 min at 21,952 g to precipitate and remove the biomass. In the supernatant solution, 1 M H_2_SO_4_ was added to reach pH 2, and the samples were placed at 4 °C overnight. Following centrifugation at 4 °C and maximum speed for 30 min (Centrifuge 5810 R, Eppendorf, Hamburg, Germany), the acidified precipitate was redissolved with CHCl_3_/MeOH: 2/1 (v/v). Following incubation at 30 °C for 15 min, the BSF solution was centrifuged at 4 °C and maximum speed for 30 min (Centrifuge 5810 R, Eppendorf, Hamburg, Germany). The supernatant was discarded, and the BSF was left to dry and, finally, stored at − 20 °C.

### Chemical oxygen demand (COD) analysis

Following the completion of each experiment, the cultures were centrifuged for 30 min at 21,952 g to precipitate and remove the biomass. The method for the determination of COD was based on instructions by a kit (Supelco Inc., Sigma-Aldrich, St. Louis, MI, USA) with COD range between 500 and 10,000 mg/L. Sample preparation, incubation, and COD quantification were performed following the kit instructions and according to Varnava et al. (2024a). The control contained the slightly modified M9 minimal medium and 1% (v/v) OW.

### Characterization of biosurfactants with FTIR

The FTIR analyses were carried out using a Bruker (Mannheim, Germany) Vertex 70 FTIR spectrometer. The spectra were acquired in the attenuated total reflectance (ATR) mode with a PIKE MIRacle ATR accessory configured with a single reflection ZnSe crystal. The BSFs were analyzed according to Tsipa et al. (2021a).

### Fly food preparation and maintenance

The *Drosophila melanogaster* wild-type, red-eyed fly strain Oregon R (OR) and a visually distinguishable, white-eyed mutant fly stain (*w*^1118^) were reared in 50 mL fly food bottles. Food recipe: 10 g agar, 24 g sugar, 43.6 g cornmeal, 30 g yeast per liter of ddH_2_O, supplemented with 3.8 mL/L propionic acid (Sigma-Aldrich, Darmstadt, Germany) and 5.6 mL/L Tegosept (20%, Sigma-Aldrich Darmstadt, Germany). The flies were flipped every 4 days in a new bottle with fresh fly food at 25 °C.

### Fly attraction to bacterially contaminated food

Two custom 50 mL falcon tube traps were prepared for each choice experiment as previously described (Kapsetaki et al. 2014). The tubes had a hole of ~ 1.2 mm in diameter, in the middle of their thermally cut and inverted conical tips. They contained ~ 1 mL of yeast paste made of 3 g of live yeast in 8 mL of ddH_2_O, which was placed inside the screw-cup of each 50 mL falcon. On the top of the yeast paste, 100 µL of an over day culture at OD_600nm_ = 5 of each bacterial strain was added. One hundred microliters of sterile LB medium was added on the yeast paste as the control.

The two traps were placed, screw-cup facing down, in a 1-L glass jar. One hundred female OR flies, 4–5 days old, were briefly anaesthetized with CO_2_, added in the jar, and covered with aluminum foil.

Each jar was incubated at 25 °C for 24 h letting the flies choose between the LB control and the bacterial culture containing fly trap. Then, flies were enumerated: the flies remaining outside the traps were enumerated upon anesthesia via CO_2_ and accounted for up to ~ 10% of the total. Flies found in each of the two traps were also measured and statistically compared.

### Fly survival to infection by feeding

1.5 mL of an over day culture of OD_600nm_ = 3 was centrifuged at 8000 g for 2 min. The bacterial pellet was redissolved in 15 mL of infection mixture containing 1.5 mL of fresh LB broth, 3 mL 20% (w/v) sucrose, and 10.5 mL ddH_2_Ο. For each of the 7 bacterial strains, 30 newly hatched OR female flies were transferred every day for 4 days on fresh fly food containing preservatives to eliminate *Drosophila* gut microbiota that grow on LB plates, then starved for 5–6 h and distributed evenly in each of the three vials containing a cotton ball soaked with 5 mL of the infection mixture. The vials were transferred at 25 °C, and survival was measured every day until all flies are dead. Two independent experiments were performed for each bacterial strain.

### Fly colonization and contagion

To test the ability of each bacterial strain to colonize and spread from fly to fly, female OR flies were fed with each bacterial strain for 24 h and let to interact in a closed space for another 24 h with females *w*^*1118*^ flies. More specifically, newly hatched OR female flies were transferred every day for 4 days on fresh fly food containing preservatives to eliminate *Drosophila* gut microbiota that grow on LB plates, then starved for 5–6 h, and let feed for 24 h on infection mixes, each of which contained a pellet of 1.5 mL over day bacterial culture (OD_600nm_ = 3) centrifuged at 8000 g for 2 min and redissolved in 15 mL of 12 mL ddH_2_O and 3 mL 20% sucrose. Following infection with each of the bacterial strains, 12 infected OR flies were transferred for 24 h into a custom 50 mL falcon tube bearing 12 holes (1.2 mm in diameter) on the lid for access to food and multiple 0.9 mm in diameter holes around it for aeration. At the same time, 12 uninfected, *w*^*1118*^ female flies, which have previously starved for 5–6 h, were added to each tube. Flies were able to reach their food only through the holes of the lid. The food was added on a Whatman filter paper disc soaked with 200 µL of 4% sucrose in water, which was placed on the outside of the falcon tube lid and covered with parafilm. Three replicates/tubes were used for each bacterial strain.

### Quantification of bacteria inside the flies

After 24 h of feeding on a bacterial strain or upon another 24 h of interaction with infected flies, three female OR and three _*W*_^*1118*^ female flies, respectively, were externally sterilized by brief dipping into 100% ethanol and air drying on a napkin and grinded via vortexing for 10 min using a 3-mm metal bead in 1.5-mL Eppendorf tubes containing 200 µL phosphate-buffered saline (PBS) (Sigma-Aldrich, Darmstadt, Germany) solution in triplicates. Eight hundred microliters of PBS was then added in each fly lysate, and 50 µL of the final mixture was spread on LB agar plates. Control uninfected flies do not show essentially any bacterial colonies on LB plates under these conditions. The plates were incubated at 37 °C for 16 h, colonies were enumerated, and colony forming units (CFUs) per fly were calculated.

### Fly BSF and OW toxicity assay

For every bacterial strain, 50 mg of BSFs produced by that strain was dissolved in 1 mL of 5% methanol in distilled H_2_O. The BSF toxicity assessment mix was prepared using 0.1 mL of the BSF solution (or 5% methanol in distilled H_2_O as control) mixed with 0.2 mL sucrose (20% in distilled H_2_O), 3.8 µL propionic acid (Sigma-Aldrich, Darmstadt, Germany), and 5.6 µL Tegosept (20% in pure ethanol, Sigma-Aldrich, Darmstadt, Germany) and 0.7 mL distilled H_2_O. That is, each BSF toxicity assessment mix contained 5 mg/mL of BSF in the solution.

To prepare 1 mL of 1, 2, and 5% OW toxicity assessment mix, 10, 20, and 50 µL of OW (or equal amount of distilled H_2_O for control) were mixed with 0.2 mL sucrose (20% in distilled H_2_O), 3.8 µL propionic acid (Sigma-Aldrich, Darmstadt, Germany), 5.6 µL Tegosept (20% in ethanol, Sigma-Aldrich, Darmstadt, Germany), and 0.8 mL distilled H_2_O.

For each toxicity assay, triplicates of 10 newly hatched OR female flies were transferred every day for 4 days on fresh fly food, then starved for 5–6 h and transferred into custom 50-mL falcon tubes with 12 holes (1.2 mm in diameter) on the lid and with multiple 0.9 mm in diameter holes all around. Flies were able to feed on the toxicity assessment mix through the holes of the lid. Two hundred microliters of the mix was used to completely soak a Whatman filter paper disc (Grade 3, 23 mm diameter) placed on the lid and covered with parafilm. Two hundred microliters of ddH_2_O was added every day on each disc to replenish the evaporated water, and parafilm was replaced.

### Statistical analysis

One-way analysis of variance (ANOVA) was performed using Prism (https://www.graphpad.com/) to determine the significance of differences in the CFUs among bacterial strains. Log-rank test was used to compare the distributions of two strains at a time regarding survival to infection by feeding. For each choice experiment, the chi-square test was performed to assess the difference of the observed ratio from an expected 1:1 ratio of flies within the two traps (bacterial culture containing vs LB control fly trap). The significance was accepted at *p* < 0.05.

## Results

### Phylogenetic tree analysis

**Fig. 1:**
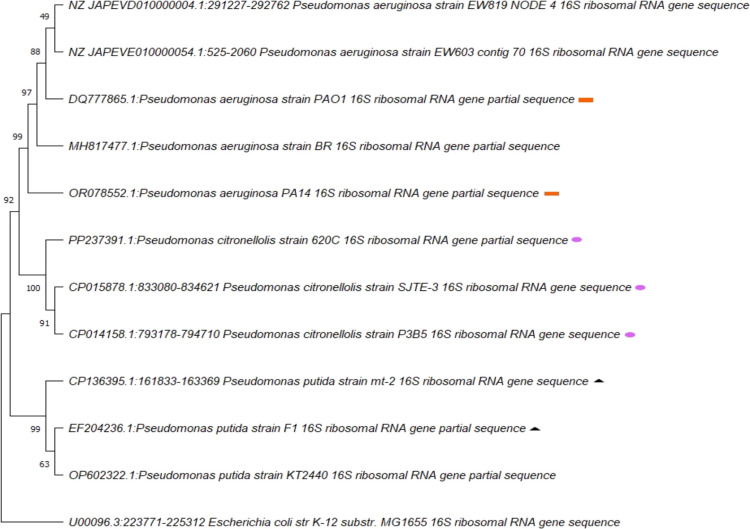
Phylogenetic tree of bacterial strains via neighbor-joining analysis, showing the position of the three different *P. citronellolis* strains studied among other *P. putida* and *P. aeruginosa* strains. This analysis is based on partial 16S rRNA gene sequences. Bootstrap values (expressed as percentages of 1000 replications) are presented at the branch points. Orange rectangle: the *P. aeruginosa*, purple oval: the *P. citronellolis*, black triangle: the *P. putida* strains studied in the present study

The phylogenetic tree of Fig. 1 shows the evolutionary relationship of the *P. aeruginosa* PAO1 and PA14; *P. putida* mt-2 and F1; *P. citronellolis* P3B5, SJTE-3, and 620C, which are the focus of this study, along with additional *P. putida* and *P. aeruginosa* strains. All the above-named strains are commonly referenced as typical of the corresponding species, but *P. citronellolis* 620C, which is recently described by us (Tsipa et al. 2021a; Varnava et al. 2024a, b). Accordingly, SJTE-3 and P3B5 cluster together suggesting that they share a common evolutionary ancestor more recently than with any other strain in the tree. The bootstrap value supporting this clade is 91, indicating a high level of statistical support for this grouping. Additionally, despite the very close evolutionary proximity of 620C with the two aforementioned strains, it appears more distant from them.

Similarly, the *P. putida* strains of interest, mt-2 and F1, despite their proximity, did not cluster together. F1 is clustered together with another *P. putida* strain, the KT2440 with a bootstrap value of 63. Finally, the *P. aeruginosa* strains, PAO1 and PA14, although related, clustered separately from each other and the other three *P. aeruginosa* strains in this tree, owing to the significant genomic variability observed among strains of this species (Panayidou et al. 2020; Lee et al. 2006).

### COD removal and BSF production for the seven strains

**Fig. 2:**
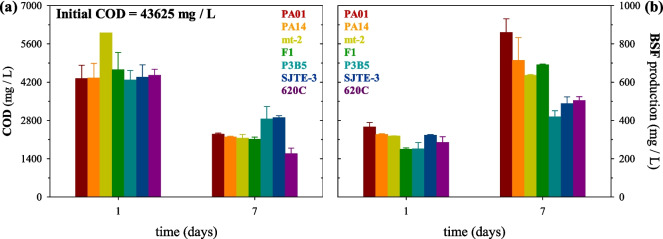
(**a**) COD remaining and (**b**) BSF production upon *Pseudomonas* strains growth on oily wastewater after 1 and 7 days

The initial COD of the OW stream was 43,625 mg/L. After 24 h (day 1) of OW biotreatment, COD removal was remarkable with the remaining COD being less than 6000 mg/L and the % COD removal > 86%, for all *Pseudomonas* strains (Table 1, Fig. 2a). *P. aeruginosa* PAO1 and PA14 exhibited comparable remaining COD and % COD removal. The remaining COD for *P. putida* F1 was lower compared to mt-2. Among the *P. citronellolis* strains, the remaining COD and % COD removal by all strains were similar.

**Table 1:** % COD removal by *P. aeruginosa* PAO1 and PA14; *P. putida* mt-2 and F1; *P. citronellolis* P3B5, SJTE-3, and 620 C after 1 and 7 days of OW biotreatment

*Pseudomonas* strains		% COD removal	
		1 day	7 days
*Pseudomonas aeruginosa*	**PAO1**	90.1	94.7
	**PA14**	90.0	95.0
*Pseudomonas putida*	**mt-2**	86.2	95.1
	**F1**	89.3	95.2
*Pseudomonas citronellolis*	**P3B5**	90.2	93.5
	**SJTE-3**	90.0	93.4
	**620 C**	89.8	96.4

After 24 h (day 1) of OW biotreatment, BSFs were effectively produced (Fig. 2b). BSFs produced by *P. aeruginosa* PAO1 were higher compared to PA14. A high amount of BSF was produced by *P. putida* mt-2 than by strain F1. Among the *P. citronellolis* strains, SJTE-3 produced the highest amount of BSF followed by 620C and then P3B5. Thus, on day 1, *P. aeruginosa* PAO1 produced the highest amount of BSF, but COD removal was similar for all the strains, except for *P. putida* mt-2, which exhibited the least COD removal.

After 7 days of OW biotreatment, COD removal was increased by all strains (Table 1, Fig. 2a). The remaining COD and % COD removal were similar among strains with the exception of *P. citronellolis* P3B5 and SJTE-3, which exhibited the least % COD removal (Fig. 2a, Table 1). The remaining COD by *P. aeruginosa* PAO1 and PA14 and, thus, % COD removal by both strains were similar and enhanced by 4.6% (PAO1) and 5% (PA14). The remaining COD and % COD removal by *P. putida* mt-2 and F1 were similar and enhanced by 8.9% and 5.9%, respectively. Among the *P. citronellolis* strains, the remaining COD and % COD removal by SJTE-3 and P3B5 strains were similar and enhanced by 3.4% and 3.3%, respectively. The remaining COD and % COD removal by 620C were lower and higher, respectively, and enhanced by 6.6%.

After 7 days of OW biotreatment, BSF production was significantly increased by all strains compared to day 1 (Fig. 2b). BSF production by the two *P. aeruginosa* strains was again higher compared to the other strains. BSFs produced by *P. aeruginosa* PAO1 increased by 135% and were similar to *P. aeruginosa* PA14, which increased by 119%*.* BSF produced by *P. putida* mt-2 and F1 increased by 100% and 178%, respectively. The amount of BSF produced by F1 was higher than that produced by mt-2, showing that over time, F1 can also efficiently produce BSF. Among the *P. citronellolis* strains, SJTE-3 and 620C produced similar amounts of BSF, followed by P3B5, which were increased by 51%, 76%, and 66%, respectively. Interestingly, the COD removal achieved by the *P. citronellolis* 620C strain was not only the highest among *P. citronellolis* strains but also the highest among all studied strains, even though the amount of BSF produced by this strain was not the highest among the strains as seen.

### Characterization of the biosurfactants extracted by the *Pseudomonas spp*.

FTIR spectroscopy was employed to characterize the BSFs produced by the different *Pseudomonas* strains. The ATR-FTIR spectra revealed distinct functional groups, providing insight into their chemical composition and classification. The BSF from *P. aeruginosa* PAO1 exhibited hydroxyl, -OH (broad stretch at 3400–3200 cm^−1^); carboxyl, -COO^−^ (C = O stretching at ~ 1720 cm^−1^); and ester, C–O–C (C-O stretching at ~ 1300–1100 cm^−1^) bands, as well as glycosidic bond (C–O–C from sugars at ~ 1000 cm^−1^) vibration, all characteristic of rhamnolipids, along with aliphatic -CH, -CH_2_, and -CH_3_ (C-H at 2960, 2930, and 2870 cm^−1^) stretches, originated from the lipid portion of BSFs (Fig. S1, Supplementary Material). These results are in agreement with previous reports on rhamnolipid-producing *P. aeruginosa* strains (Müller et al. 2010; Soberón-Chávez et al. 2021). The lipid content was similar for all the BSFs studied herein. *P. aeruginosa* PA14 displayed these rhamnolipid-associated peaks but also showed amide I (C = O stretching at 1650 and 1625 cm^−1^) and amide II (out-of-phase combination of the N–H in plane bend and the C-N stretching vibration at 1530 cm^−1^) vibrations of the peptide bond, suggesting the more complex structure of a glycolipopeptide-type BSF (Liu et al. 2018; Haque et al. 2020) (Fig. S2, Supplementary Material). *P. putida* mt-2 also exhibited amide bands, supporting the presence of a glycolipopeptide (Fig. S3, Supplementary Material). *P. putida* strains are known for their ability to produce diverse BSFs which is evident upon assessing the BSFs originated by *P. putida* F1. This BSF lacked amide bands, but the presence of lipid-related C-H stretching (2960, 2930, and 2870 cm^−1^), carboxyl (~ 1720 cm^−1^), ester (~ 1300–1100 cm⁻^1^), and glycosidic (~ 1000 cm^−1^) bands suggested a glycolipid structure (Fig. S4, Supplementary Material), consistent with earlier findings on glycolipid-producing *P. putida* strains (Tuleva et al. 2002; Kuiper et al. 2004). The BSF from *P. citronellolis* P3B5 and SJTE-3 displayed the characteristic vibrations of a glycolipid-type BSF (Fig. S5, 6 Supplementary Material). Lastly, BSF produced by *P. citronellolis* 620C was characterized in detail before by us and confirmed as a lipopeptide (Tsipa et al. 2021a; Varnava et al. 2024a).

### Fly attraction to bacterially contaminated yeast paste

**Fig. 3:**
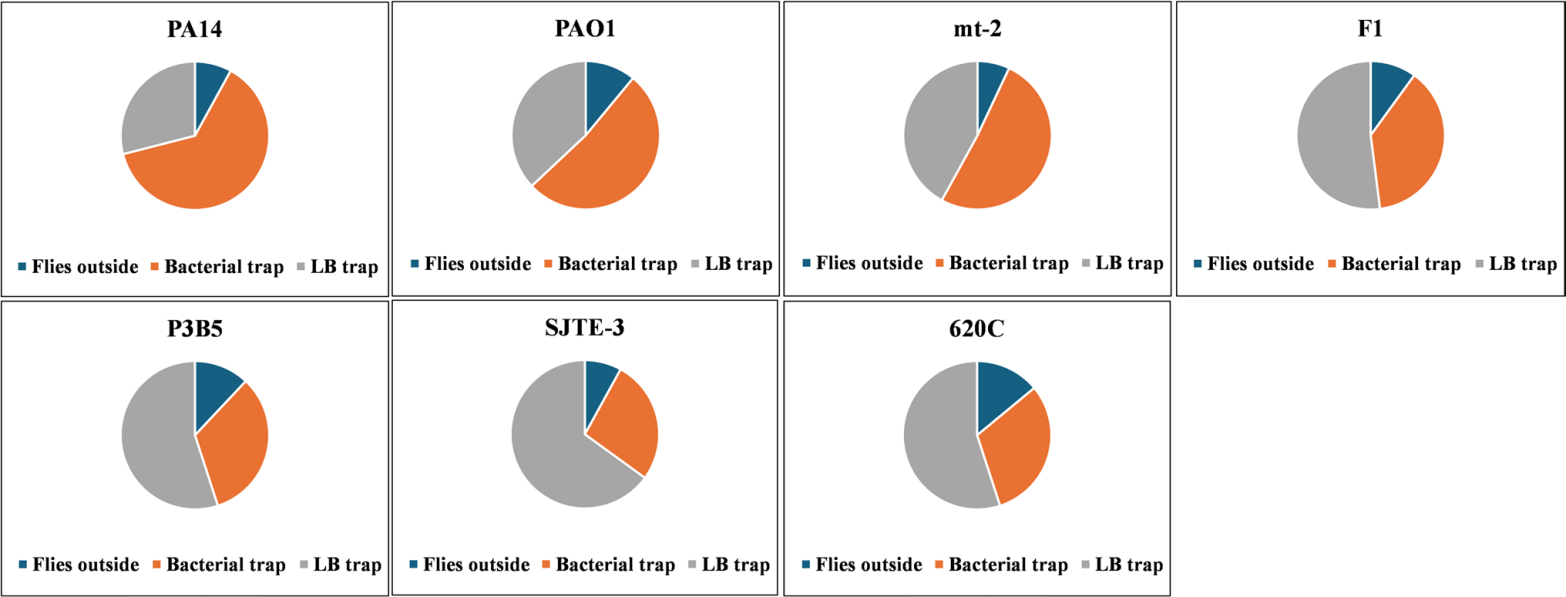
Pie charts showing percentages of flies remaining outside (blue) and those entering the trap containing bacterial culture (orange) and the control LB media trap (gray). Each chart represents two independent experiments summing up to *n* = 200 female OR flies for each strain, except PA14 (*n* = 199 flies) and F1 (*n* = 198 flies)

Firstly, the attraction of the flies to each of the bacterial strains was evaluated to examine if the flies would choose as food the yeast paste that contained bacterial cultures grown in LB over the one that contained control LB medium. After 1 day, the flies outside and inside of each trap were measured (Fig. 3), and the numbers of flies in the two paired traps were compared by a chi-square test (Table 2). Accordingly, flies were more attracted to *P. aeruginosa* PA14 (2.2:1), followed by *P. aeruginosa* PAO1 (1.4:1) and *P. putida* mt-2 (1.2:1). The flies were repelled by *P. putida* F1 (0.73:1), but more so from *P. citronellolis* strains, SJTE-3 (0.41:1), 620C (0.56:1), and P3B5 (0.6:1) (Table 2).

**Table 2:** Attraction (positive) or repulsion (negative) of flies for the bacterial media containing trap vs LB containing trap. Chi-square score was calculated based on the observed number of flies in each of the two traps versus an expected even distribution. Differences are accepted as significant at *p* = 0.05, if *χ*^2^ > 3.84 (d.f. = 1). PA14, PAO1, and mt-2 exhibited significant attraction, while the remaining four exhibited significant repulsion (attraction negative)

Bacterial strains	Flies’ attraction by contaminated food	Chi-square score
*P. aeruginosa* PA14	Positive	141.8
*P. aeruginosa* PAO1	Positive	99.4
*P. putida* mt-2	Positive	95.6
*P. putida* F1	Negative	97.6
*P. citronellolis* P3B5	Negative	110.6
*P. citronellolis* SJTE-3	Negative	151.5
*P. citronellolis* 620 C	Negative	113.6

### Fly colonization assay and contagion assessment

**Fig. 4:**
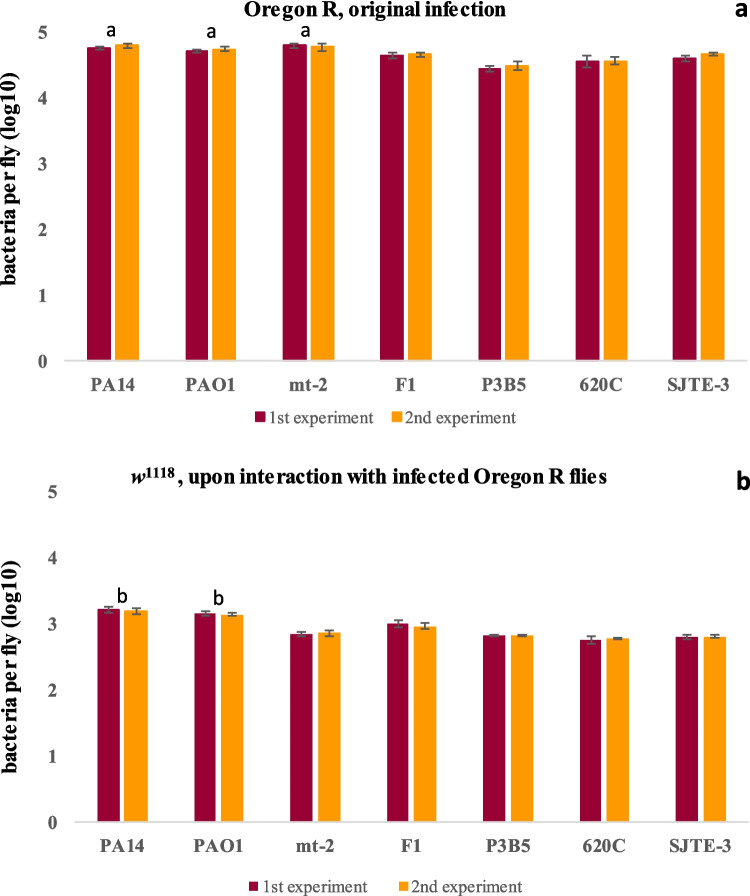
CFUs per fly of Oregon R flies, originally infected for 24 h (**a**), and of *w*^1118^ flies, upon 24-h interaction with infected Oregon R flies (**b**). For each of the two replicate experiments, triplicates of three flies were used to calculate log10 bacterial CFUs per fly. ANOVA test was performed by combining the results of both experiments. In each plot, statistically significant differences (*p* < 0.0001) are indicated between the labeled, as a or b, and the non-labeled strains

Microbiological analyses of infected OR (Fig. 4a) and *w*^*1118*^ flies (Fig. 4b) followed to assess internal bacterial load. The colonization of OR flies varied between 4.45 and 4.79 log10 CFUs per fly, indicating a marginally higher ability of *P. aeruginosa* strains and *P. putida* strain mt-2 to colonize the flies (Fig. 4a, ANOVA *p* < 0.0001, Tuckey’s post hoc test).

Contamination of externally sterilized *w*^*1118*^ flies aimed to assess contagion via the intestinal route. CFUs varied between 2.77 and 3.22 log10 per fly and were marginally higher for *P. aeruginosa* strains and *P. putida* strain F1 to colonize the flies (Fig. 4a, ANOVA *p* < 0.0001, Tuckey’s post hoc test). Thus, *P. citronellolis* strains exhibited marginally but significantly less ability to colonize and spread from fly to fly.

### Fly survival assay and microbiological analyses following feeding on the different *Pseudomonas* strains

**Fig. 5:**
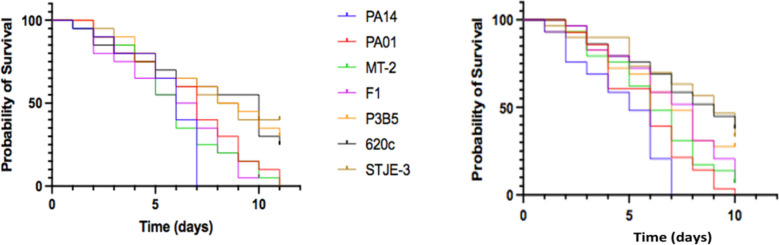
Kaplan–Meier survival plots of the percentage of live flies feeding on one of the seven bacteria strains. Left and right plots show independent experiments done under the same conditions. Each line in each graph is represented by 30 female OR flies fed with one of the seven bacterial strains. Flies’ survival was measured up to 11 days

To assess *Drosophila* virulence following feeding on *Pseudomonas* strains, triplicates of 10 OR female flies were fed with a bacterial infection mix. Under our experimental conditions, uninfected flies can live more than 20 days (Panayidou et al. 2020; Charalambous et al. 2022). Flies dying from infection were measured every day for at least 10 days. Kaplan–Meier survival plots indicate strain-to-strain differences based on two independent experiments (Fig. 5). The time required for 50% or 100% of flies to die (LT50 and LT100, respectively) is shown on Table 3. *P. aeruginosa* strain PA14 was the most virulent, killing all the flies within 7 days. *P. aeruginosa* strain PAO1 and the two *P. putida* strains were intermediate in virulence killing all or essentially all flies between 10 and 11 days, while the three *P. citronellolis* strains were the least virulent, leaving at least 30% survivors by day 10 (Fig. 5 and Table 3).

**Table 3:** *Pseudomonas* strains ranked in order of fly mortality rate (LT50) upon feeding on virulent infection mixes. LT50 and LT100 of 60 flies for each bacteria strain. Log10 of CFUs per fly, ± standard deviation, upon feeding on the bacterial infection mix of each *Pseudomonas* strain for 24 h. In each CFU assessment experiment, triplicates of three female OR flies were fed with one of the seven bacterial strains and for 24 h

Bacterial strains fed to flies	LT50 (days)	LT100 (days)	CFU per fly (log10)
*P. citronellolis* 620C	9.5	> 11	4.6 ± 0.2
*P. citronellolis* SJTE-3	8.5	> 11	4.7 ± 0.1
*P. citronellolis* P3B5	8	> 11	4.4 ± 0.1
*P. putida* F1	7	11	4.5 ± 0.3
*P. aeruginosa* PAO1	6.5	10.5	4.8 ± 0.2
*P. putida* mt-2	6	11	4.9 ± 0.1
*P. aeruginosa* PA14	5.5	7	4.8 ± 0.3

Similarly to the bacterial colonization assays, at 24 h of feeding on virulent infection mixes, the colonization of OR flies varied between 4.35 and 4.85 log10 CFUs per fly and indicated a marginally higher ability to *P. aeruginosa* strains and *P. putida* strain mt-2 to colonize the flies compared to *P. citronellolis* strains (ANOVA *p* < 0.0026, Tuckey’s post hoc test).

### Fly survival assay following feeding on BSFs extracted from *Pseudomonas* strain cultures in minimal media containing oily wastewater

**Fig. 6:**
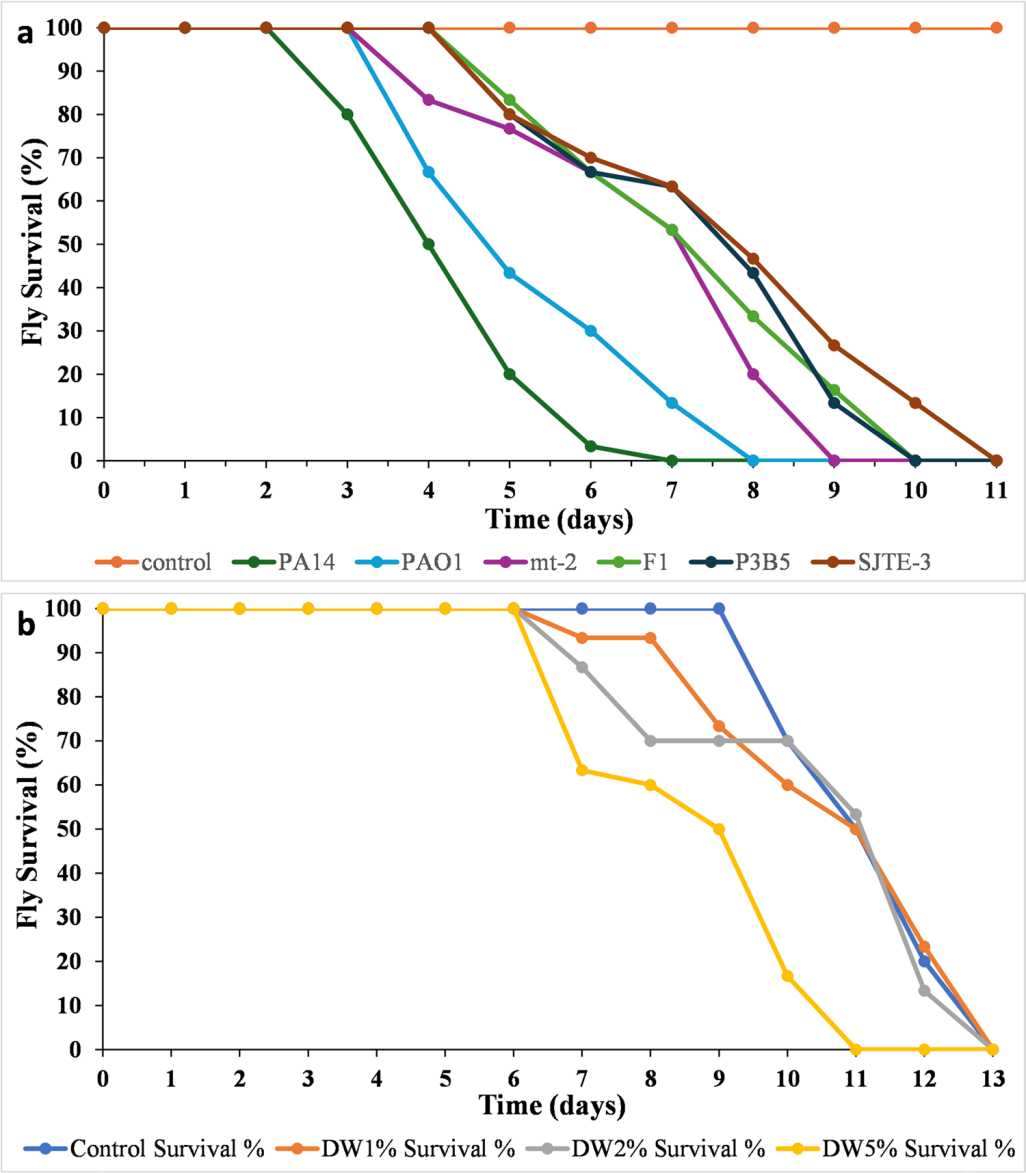
Percentage of survival over time of flies feeding on (**a**) bacterially produced BSFs grown for 7 days in 1% OW and (**b**) 1, 2, or 5% of OW or vehicle control medium. For each curve, 30 female OR flies were used

To assess the relative toxicity of bacterially produced BSFs, we fed female OR flies with a toxicity assessment mix containing BSF. All vehicle fed flies lived for more than 11 days (Fig. 6). Flies fed with BSFs of the *P. aeruginosa* strains PA14 and PAO1 killed all the flies within 6.8 and 8 days, respectively. BSFs from *P. putida* strains mt-2 and F1 killed all flies within 9 and 10 days, respectively. However, BSFs from *P. citronellolis* strains, P3B5 and SJTE-3, killed all flies within 10 and 11 days, respectively (Fig. 6 and Table 4). Overall, *P. aeruginosa* and *P. citronellolis* strains exhibited, respectively, the lowest and the highest LT50 and LT100.

**Table 4:** Average LT50 and LT100 of 30 flies fed on each BSF and with 1, 2, and 5% of DW. BSFs are ranked according to their toxicity (least to highest LT50 and LT100)

Species-producing BSF on OW	Strain-producing BSF on OW	Fly LT50 (days)	Fly LT100 (days)
*P. citronellolis*	SJTE-3	8.5	11
	P3B5	8.25	10
*P. putida*	F1	8	10
	mt-2	8	9
*P. aeruginosa*	PAO1	5	8
	PA14	4	6.8
	**Concentration of DW**		
	DW (1%)	11	13
	DW (2%)	11.2	13
	DW (5%)	9	11

### Fly survival assay following feeding with oily wastewater

To assess the toxicity of the OW, that is DW, on *Drosophila*, three different concentrations of the OW, 1, 2, and 5% were assessed. The alive flies were measured every day for 14 days, when all flies were dead. Survival curves were plotted (Fig. 6), and LT50 and LT100 were also calculated. The highest DW concentration used (5%) exhibited an LT50 between 8 and 11 and an LT100 between 11 and 12, while 1% and 2% of DW exhibited very similar profiles with an LT50 between 11.5 and 13 and an LT100 of 13 (Table 4). Given that vehicle-fed (4% sucrose) flies in this assay have an LT50 of ~ 10 days, the DW used exhibits low to no acute toxicity in flies.

## Discussion

The main aim of this study was to evaluate key *Pseudomonas* species and disparate strains for their virulence and the toxicity of the BSFs they produce upon growth on OW in *Drosophila*. The phylogenetic tree analysis showed that, although *P. citronellolis* 620C draft genome exhibits high DNA sequence homology with *P. citronellolis* P3B5 and SJTE-3 genomes, as shown by BLAST analysis (Tsipa et al. 2021a), *P. citronellolis* strains SJTE-3 and P3B5 are closer to each other. Similarly, *P. aeruginosa* PAO1 and PA14 are in different branches. In addition, *P. putida* mt-2 and F1 do not cluster together. Therefore, even strains of the same species are evolutionarily distinct.

Interestingly, all strains can effectively degrade OW to produce BSFs after 24 h, but *P. aeruginosa* strain PAO1 seems to produce more BSFs than any other strain. However, the COD removal, indicative of OW treatment, among all species was similar reaching 90% except for *P. putida* mt-2, which was slightly lower. BSFs enhance the biodegradation of hydrophobic compounds such as hydrocarbons, which are included in OW streams (Matsumiya et al. 2007; Affandi et al. 2014). As expected, longer incubation times (7 days) allowed all strains to produce more BSFs and improve COD removal. However, among *P. citronellolis* strains, there is a difference in COD removal efficiency. In particular, 620C resulted in a higher one, which was also the highest among all strains, showing greater efficiency in degrading this type of OW, whereas SJTE-3 and P3B5 achieved similar COD removal. One explanation for this could be the fact that *P. citronellolis* 620C was isolated using activated sludge and the OW used in this study. Therefore, the strain may be adapted to it (Tsipa et al. 2021a) having been effectively utilized in several biodegradation applications of this type of OW (Varnava et al. 2024a, b). Another explanation could be that this strain stands out genetically among the three *P. citronellolis* strains compared, as seen in the phylogenetic tree; thus, it may have metabolic properties which lead to more effective OW degradation. A genomic analysis including more than just the 16S rRNA would provide more insights into the differences among *P. citronellolis* strains.

The use of low-cost substrates, such as industrial wastewater, is an effective way to reduce the cost of BSF production which, currently, hampers its broad use as an alternative to synthetic surfactants (Varnava et al. 2024a). Furthermore, this is a circular bioeconomy concept where wastewater is used to form added-value products, the BSFs, which also contribute to wastewater biodegradation. Towards this direction, OW biotreatment seems a promising solution, while *Pseudomonas* strains are promising bioagents as confirmed by the present study.

The first evaluation assay using *Drosophila* was the attraction of the flies to cultures of the different strains. Both *P. aeruginosa* strains and *P. putida* mt-2 attract the flies to a great extent, while *P. putida* F1 and all *P. citronellolis* strains repel them*.* Certain strains may generate volatile organic compounds (VOCs) upon growth on LB that attract the flies (Tomberlin et al. 2017; Kapsetaki et al. 2014). This may be the case in *P. aeruginosa* strains which are known toxic VOC producers, such as 2-acetoaminophenone (Filipiak et al. 2012; Shestivska et al. 2012; Kapsetaki et al. 2014), and probably *P. putida* mt-2, whereas *P. putida* F1 and all the *P. citronellolis* strains might produce no such VOCs, thus being less attractive to flies. The fact that mt-2 and F1 attract the flies differently can also be explained by their distance in the phylogenetic tree which indicates that they may have alterations in their metabolism.

Regarding colonization, it is known that *P. aeruginosa* strains can colonize flies (Apidianakis & Rahme 2009; Mulcahy et al. 2011; Kapsetaki et al. 2014; Panayidou et al. 2020; Charalambous et al. 2022), which was also evident in the current study. *P. putida* F1 is third in the row of colonization ability. However, contagion to uninfected *w*^*1118*^ flies by *P. putida* mt-2 and *P. citronellolis* SJTE-3, P3B5, and 620C was marginally less but effective. Thus, *P. citronellolis* strains colonize less and spread less from fly to fly. *Pseudomonas* species have remarkable metabolic versatility, reflected by their ability to colonize numerous ecological niches. However, this ability could also be virulent against plants, insects, and nematodes (Matthijs et al. 2009). In the current study, *P. aeruginosa* strains were the most virulent, followed by *P. putida* and, then, *P. citronellolis* strains. It is well-established that *P. aeruginosa* is virulent to flies when injected into the body cavity (Panayidou et al. 2020). However, the virulence of *P. putida* to flies was not known. This is the first study showing evidence of *P. putida* virulence in flies. *P. putida* is closely related to *entomophila* which is a well-known entomopathogen (Matthijs et al. 2009) affecting *Drosophila melanogaster* and other insects (Vodovar et al. 2006). *P. entomophila* is highly virulent for both *Drosophila* larvae and adults by oral ingestion (Vodovar et al. 2005). Although *P. putida* may be a pathogen in a clinical context (Fernández et al. 2015), it is well-known for its environmental resilience rather than its virulence and pathogenicity. However, under stress conditions, *P. putida* expresses stress response systems which may provoke virulence to other organisms (Wu et al. 2011; Svenningsen et al. 2015; Alzahrani et al. 2023). Therefore, the current study raises further scientific questions regarding the virulence factors and resistance genes of *P. putida* mt-2 and F1.

*P. citronellolis* is a hydrocarbon-degrading bacterium (Bhattacharya et al. 2003), able to produce BSFs (Jacques et al. 2008; Santos et al. 2008) and PHAs (Rebocho et al. 2019). However, it has been studied to a much lesser extent in environmental applications compared to the other two species. Regarding its virulence, two studies show the pathogenicity of *P. citronellolis* clinical isolates (Hwang et al. 2023; Lin et al. 2024). However, no further information about its pathogenicity is known. This is the first study showing evidence regarding virulence, showing that this species is not as virulent as *P. putida* and *P. aeruginosa*. This shows the promising potential of *P. citronellolis* use in bioremediation, agriculture, and other biotechnological applications. Indeed, studies are showing that *P. citronellolis* is a plant growth promoter (Adhikary et al. 2019, 2022; Silambarasan et al. 2020).

The virulence of microorganisms implies that these microorganisms can strategically be used as antinsecticidal agents. On the other hand, if used under non-controlled conditions, flies will be attracted and spread within the ecosystem, potentially affecting plants, animals, and aquatic life. Similarly, the toxicity of their BSFs on flies implies that if toxic, they can intentionally be used as biocontrol agents, while the less toxic ones can be used to several other applications without any detrimental effects to the environment. In this study, the toxicity of BSFs aligns with the virulence of their *Pseudomonas* spp. producers. The present study confirmed, through FTIR, that *P. aeruginosa* PAO1 produces rhamnolipids. It was determined that *P. aeruginosa* PΑ14 and *P. putida* mt-2 form glycolipopeptides, while *P. putida* F1, *P. citronellolis* P3B5, and SJTE-3 produce glycolipids, which are novel discoveries to the best of the authors’ knowledge. *P. citronellolis* 620C is known to produce lipopeptides (Tsipa et al. 2021a; Varnava et al. 2024a). These findings highlight the strain-specific nature of BSF synthesis. It is demonstrated that, even under identical culture conditions, the BSF structure is primarily governed by the bacterial strain rather than the carbon source. Therefore, this study suggests that the toxicity of BSFs is lined up with the virulence of their microbial origin. Therefore, the most toxic BSFs are the ones produced by *P. aeruginosa* strains, and the least toxic BSFs are formed by *P. citronellolis* strains. Interestingly, rhamnolipids of *P. aeruginosa* strains have been reported to be “slightly toxic” when tested in *Aliivibrio fischeri* using the Microtox acute toxicity assay (Hogan et al. 2019). In addition, their ecotoxicity when tested with *Daphnia magna* was at the same order of magnitude as that of synthetic surfactants (Varnava et al. 2024a). Regarding the ecotoxicity of (glyco)lipopeptides of *Pseudomonas* spp., there is no information to the best of the authors’ knowledge. However, the lipopeptide surfactin, formed by *Bacillus subtilis*, was more toxic to *A. fischeri* and *D. magna* compared to the lipopeptide of *P. citronellolis* 620C (Varnava et al. 2024a). In addition, rhamnolipids were reported to be more toxic to *D. magna* than surfactins, indicating the low toxicity of the lipopeptide of *P. citronellolis* 620C (Varnava et al. 2024a). Considering also that *P. citronellolis* strains (a) repel flies and (b) are the least virulent to flies and (c) their BSFs are the least toxic to flies, these strains might be safer to use in the environment for OW biodegradation and other applications.

Notably, the toxicity of 1, 2, and 5% of DW used in this study is less compared to the virulence of *Pseudomonas* strains and the toxicity of their BSFs after growth on the 1% DW media. This suggests that DW can be used to produce BSFs, and the resulting microbial culture may be used as a biocontrol agent in agriculture. Since *P. aeruginosa* strains and their BSFs exhibit higher attraction, colonization, virulence, and toxicity to flies, they may be used as bioinsecticides. Several fly species can cause substantial agricultural damage, such as *Drosophila suzukii* and the Mediterranean fruit fly.

*D. melanogaster*, used in this study, is also relevant to human disease due to evolutionary conserved features shared between *Drosophila* and vertebrates, such as signal transduction pathways, innate immune cascades, and tissue and organ physiology (Panayidou et al. 2014; Younes et al. 2020). Moreover, *Drosophila* research provides insights into microbial pathogenicity (Panayidou et al. 2014; Michael Harnish et al. 2021). Therefore, the results of the current study indicate that *P. citronellolis* is not as likely to be as pathogenic to humans as *P. aeruginosa*. However, further research is necessary to assess the pathogenicity of *P. citronellolis*, including biofilm formation and exoenzyme production, the ability to produce hemolysis, and patterns of antibiotic multi-resistance. These studies are even more significant for *P. citronellolis* 620C, which was proven to be the most innocuous among the strains studied.

## Supplementary Information

Below is the link to the electronic supplementary material.

Supplementary file 1 (PDF 161 KB)

## Data Availability

The datasets generated and/or analyzed during the current study are available from the corresponding authors, Argyro Tsipa and Yiorgos Apidianakis, upon reasonable request.
