# Supplementary material for: Drosophila attraction, colonization, contagion, and mortality by Pseudomonas spp. and toxicity of their biosurfactants
Source: Appl Microbiol Biotechnol. 2025 Jun 4;109(1):135. doi: 10.1007/s00253-025-13518-x (PMC12137457; doi:10.1007/s00253-025-13518-x)
Supplement: Supplementary file 1 — (PDF 161 KB) [file 253_2025_13518_MOESM1_ESM.pdf]

Drosophila attraction, colonization, contagion and mortality by _Pseudomonas_ spp. and toxicity of their biosurfactants

Argyro Tsipa[1,2*], Maria Pettemereidi[3], Constantina K. Varnava[1], Izel Ungor[3], Eftychia Fragkou[3], Yiorgos Apidianakis[3*]

[1] Department of Civil and Environmental Engineering, University of Cyprus, 75 Kallipoleos, Nicosia 1678, Cyprus
[2] Nireas International Water Research Centre, University of Cyprus, Nicosia 1678, Cyprus[3] Department of Biological Sciences, University of Cyprus, Nicosia 1678, Cyprus

*corresponding authors: Dr Yiorgos Apidianakis (apidianakis.giorgos@ucy.ac.cy) and Dr Argyro Tsipa (tsipa.argyro@ucy.ac.cy)

Supplementary Material

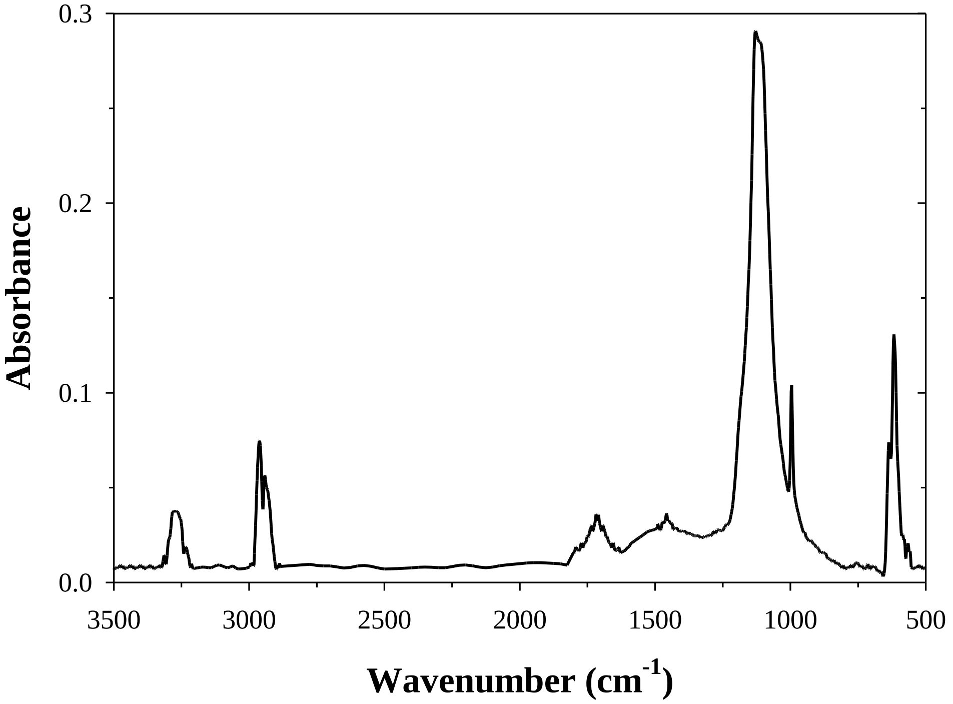

Figure S1. FT-IR spectra of the rhamno(glyco-)lipid-type biosurfactant produced by *P. aeruginosa* PAO1 when cultivated in oily wastewater.

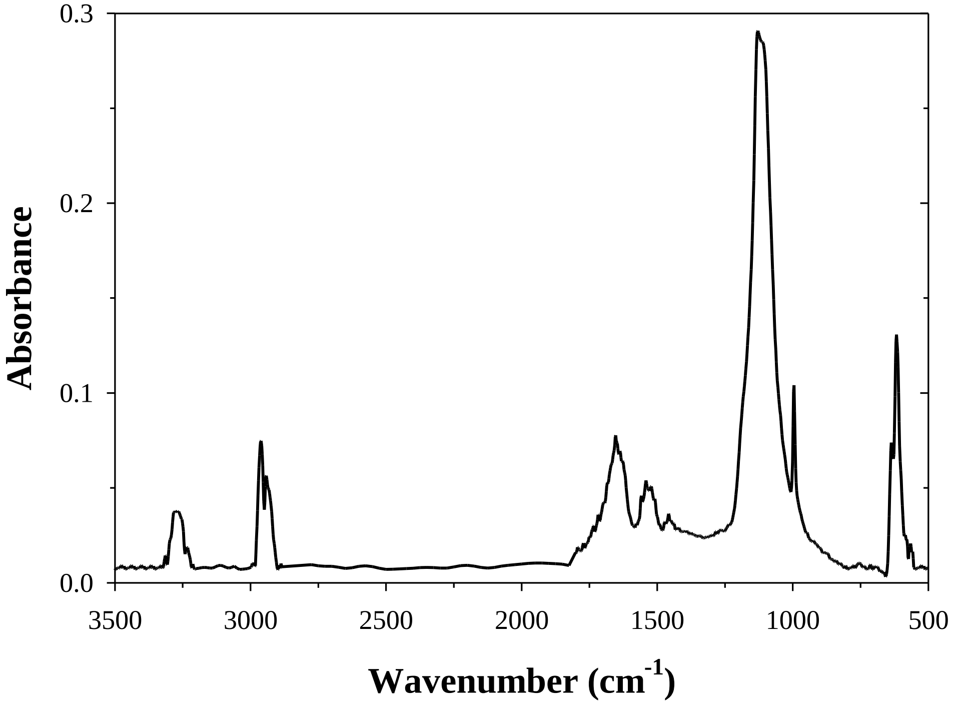

Figure S2. FT-IR spectra of the glycolipopeptide-type biosurfactant produced by *P. aeruginosa* P14 when cultivated in oily wastewater.

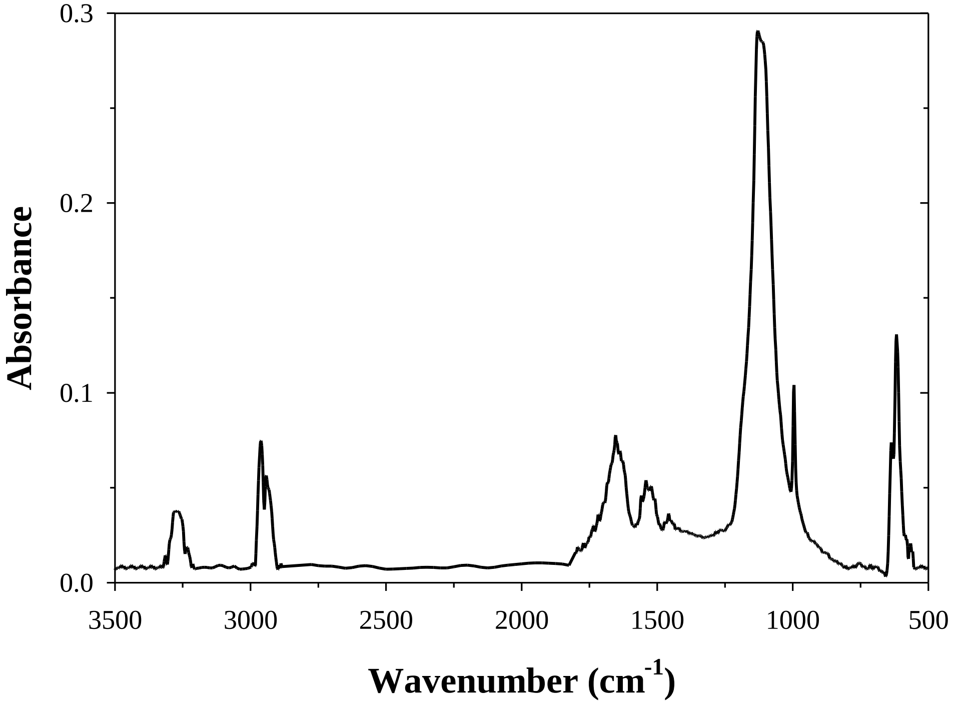

Figure S3. FT-IR spectra of the glycolipopeptide-type biosurfactant produced by *P. putida* mt-2 when cultivated in oily wastewater.

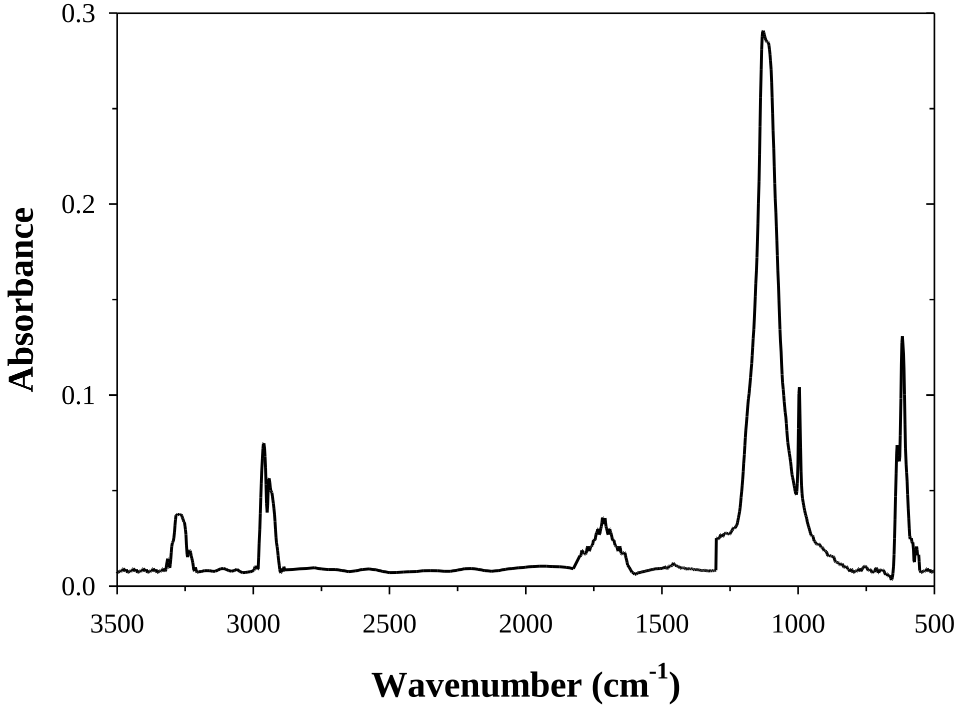

Figure S4. FT-IR spectra of the glycolipid-type biosurfactant produced by *P. putida* F1 when cultivated in oily wastewater.

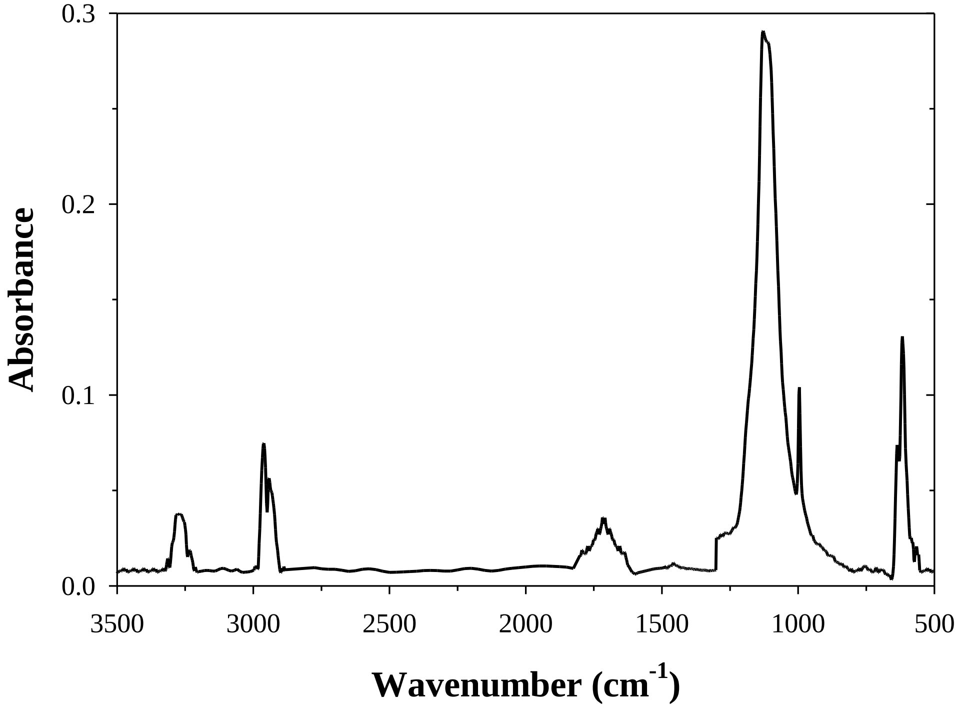

Figure S5. FT-IR spectra of the glycolipid-type biosurfactant produced by *P. citronellolis* P3B5 when cultivated in oily wastewater.

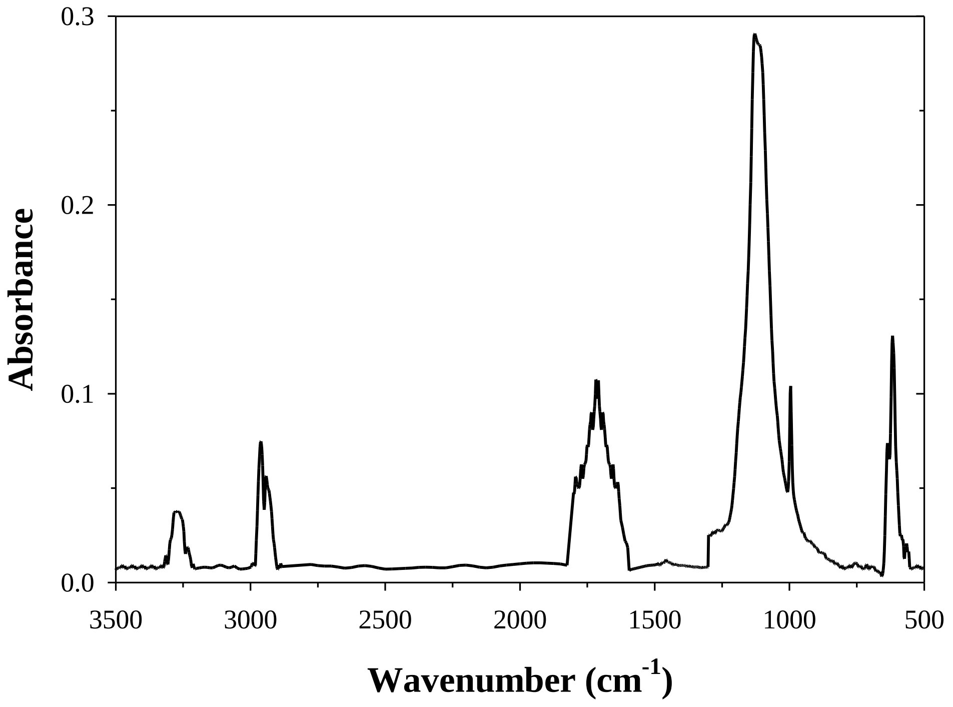

Figure S6. FT-IR spectra of the glycolipid-type biosurfactant produced by *P. citronellolis* SJTE-3 when cultivated in oily wastewater.
